# Stroke patient rehabilitation: an analysis of the effects of perturbation training combined with hip unloading gait strategies

**DOI:** 10.3389/fneur.2025.1495071

**Published:** 2025-02-21

**Authors:** Senchao Fan, Yiran Ma, Yu Pan

**Affiliations:** ^1^Department of Physical Medicine and Rehabilitation, Beijing Tsinghua Changgeng Hospital, Tsinghua University, Beijing, China; ^2^Tianjin University of Technology, Tianjin, China

**Keywords:** stroke, clinical rehabilitation, hip joint weight-reduced perturbation walking training, balance issues, walking function recovery

## Abstract

**Background:**

Perturbation training has been proven effective in reducing the risk of falls in stroke patients. When combined with hip unloading walking training, it may further enhance the restoration of walking and balance functions. This study aims to explore the rehabilitation effects of this combined training strategy on walking disabilities in post-stroke patients, with the goal of providing new methodological guidance for clinical rehabilitation.

**Methods:**

A randomized controlled trial was conducted at Beijing Tsinghua Changgung Hospital with stroke patients aged 18–70 years. Thirty patients were included and randomly assigned to three groups: the hip unloading walking group, the hip unloading plus perturbation walking group, and the control group, with 10 patients in each. The effectiveness of the training was assessed before and after using the 10-meter walk test and the Berg Balance Scale, along with secondary indicators including the Fugl-Meyer Assessment for Lower Extremity, Functional Ambulation Categories, three-dimensional gait analysis, and the timed up-and-go test.

**Conclusion:**

Compared to other types of unloading perturbation training methods, hip unloading training exhibits unique superiority. Perturbation training significantly improved the balance and walking efficiency of stroke patients, especially those with severe balance issues, outperforming traditional rehabilitation methods. The unloading group also showed a positive trend, although it did not reach statistical significance. This demonstrates the potential value of perturbation training in stroke rehabilitation.

## 1 Introduction

Stroke patients face significant challenges in walking functionality, particularly in maintaining balance control and mitigating the risk of falls. Due to neurological impairments caused by brain damage, common manifestations include reduced muscle strength, diminished coordination, and prolonged reaction times, collectively contributing to unstable gait. Typical symptoms include irregular stride lengths, uneven walking speeds, and a loss of gait symmetry. These factors not only reduce walking efficiency but also significantly increase the risk of falling (1). Additionally, impaired balance makes it difficult for stroke patients to rapidly respond to balance challenges such as quick movements or changes in direction, thereby increasing the likelihood of falls (2).

In this context, conventional walking training methods may not sufficiently enhance the walking safety and functional efficiency of stroke patients. Therefore, employing a composite rehabilitation strategy that combines unloading techniques with perturbation training is particularly important. This approach, by simulating daily life walking challenges, comprehensively strengthens the patients’ gait dynamics and balance responses, effectively enhancing balance control and significantly reducing the risk of falls, thereby improving the overall quality of life of the patients (3).

Perturbation-Based Balance Training (PBBT) is a task-specific intervention designed to improve reactive balance control after losing balance in a safe and controlled environment (4). Recent systematic reviews and meta-analyses of randomized controlled trials (RCTs) and clinical controlled trials have shown that PBBT plays a positive role in reducing the risk of falls among stroke patients (5). In one study, Pai et al. found that elderly participants reduced their risk of falls by 50% 12 months after a single session of 24 induced-slip training sessions (6). Mansfield and others also found that repeated exposure to external perturbations improved the reactive stepping response (i.e., reduced the frequency of multiple steps and foot collisions) among the elderly (7). Furthermore, in an open trial, Parkinson’s patients who participated in 10 days of perturbation training significantly improved their reactive step length, step initiation, and walking speed, and maintained these improvements after 2 months (8).

Currently, rehabilitation technologies for restoring walking function in stroke patients mainly include physiotherapy, unloading walking training, and walking rehabilitation robots (9). Physiotherapy, which involves strength training, flexibility exercises, and coordination and balance training, is adaptable and effectively enhances daily life quality. However, its effectiveness heavily depends on the patient’s continuous participation and effort, and improvements may be slow. On the other hand, walking rehabilitation robots provide precise motion guidance through wearable exoskeletons or walking simulators, suitable for early rehabilitation of patients with severe functional impairments. This method allows for high-intensity training in a safe environment but has high equipment costs and may limit patients’ opportunities for autonomous motivation, potentially impacting long-term recovery capabilities. Clinically used unloading walking training, which involves suspending the pelvis with a harness to reduce weight-bearing, has been shown to significantly improve walking function in hemiplegic patients. However, the method of suspending the pelvis partially reduces weight pressure during walking. Although this effectively reduces weight, its rigidity may interfere with the patient’s natural gait, affecting the naturalness and effectiveness of the training, leading to poor compliance and impacting its clinical application (10).

However, the hip unloading perturbation device used in this study has significant advantages in terms of focus and specificity. The hip unloading walking training device is a novel type of walking training equipment, commonly used in exoskeleton robot training methods. It changes from the fixed and suspended torso and pelvic harness unloading mode to a fixed hip-pelvic area unloading mode, acting directly on the patient’s hips to control unloading more precisely and effectively simulate muscle activity in a natural gait. This localized unloading method reduces constraints on the upper body, enhancing the comfort and naturalness of the training. By providing unloading while reducing interference with the torso, it strengthens the patient’s trunk control training, improving walking ability (11, 12).

### 1.1 Device overview

**Figure 1:**
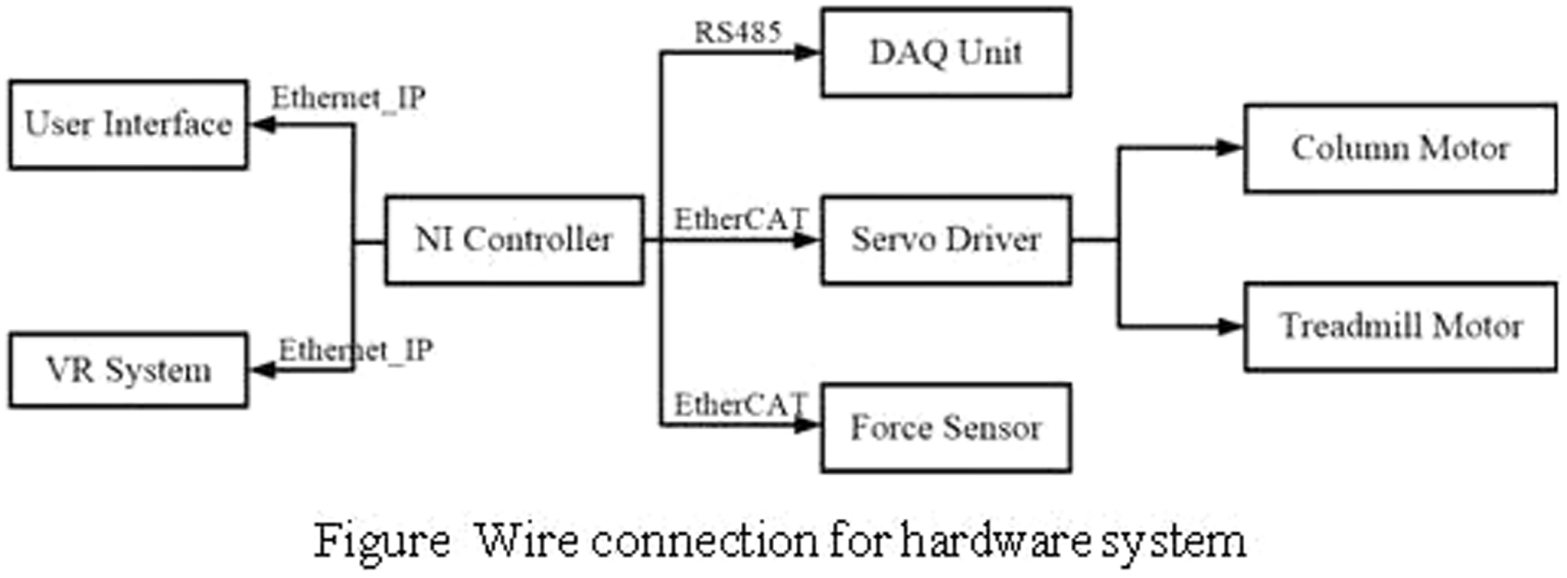
Wire connection for hardware system.

The SE-NaturaGait1® robot (Figure 1) was made up with five parts, namely Column system, Pelvis system, Operation system, treadmill and Virtual Reality (VR) system. The column system was made up by the lead screw, linear rail and servo motor. By driving a motor, which can make the lead screw move up and down in vertical direction to adjust the position to fit for the patients while training. The pelvis system was a multi-DOF mechanic system with dynamic weight loss function. The operation system provided simplify operation user interface for therapists. Multiple training modes were designed for patients’ training in different periods with treadmill system. While in the training, the patients could have interacted with VR system via the user-friendly interactive VR games. Finally, the safety protection mechanism were designed for the whole system to avoid the secondary damage for the patients during the training.

**Figure 2:**
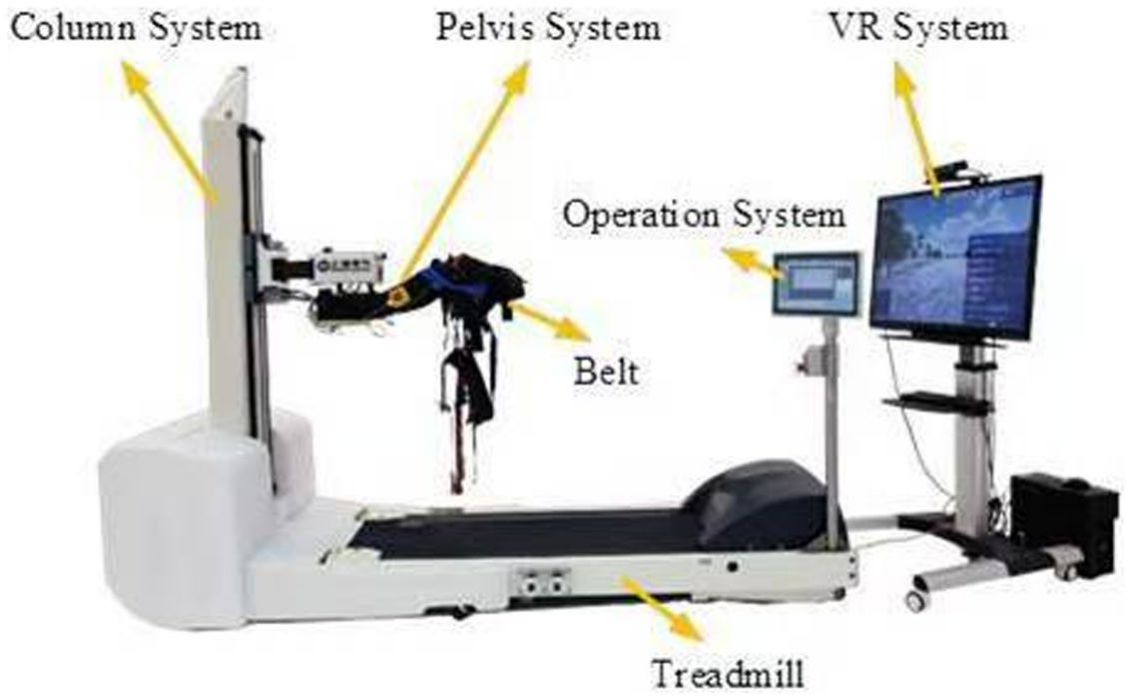
The pelvis system.

The pelvis system was connected to the column system to adjust the patients’ posture during training. The pelvis system is described in Figure 2, the foundation support is connected with the linear rail. This part was designed as parallelogram mechanism, so it could realize the pelvic left and right movement. The lateral movement can be completed around the horizontal axis and the torsion movement can be realized around the rotating axis.

Compared to some robotic system, which support the patients’ body weight by an overhead harness. NG1 allows the operator to adjust the height of the pelvic mechanism by driving the column system to support the weight of the patient. Patients do not have to be supported from above by a harness, which may lead to an uncomfortable training posture. Furthermore, some walking assistant devices cannot ensure the safety of patients while training. Overhead body weight support system limit the motion range of the trunk and pelvis. NG1 has 3 DOFs at pelvis, namely flexion-extension, lateroflexion and rotation, which interfere less.

The hardware system consists of the controller, force sensor, motor and motor driver. Two servo motors were used for column system to adjust the height and treadmill system to control the training speed. The column motor was equipped with absolution encoder for position and velocity measurement, while the treadmill motor equipped with incremental encoder. Two 6-axis force and torque sensors were installed on the both sides of pelvis system, and connected to the belt for force value acquisition during training.

**Figure 3:**
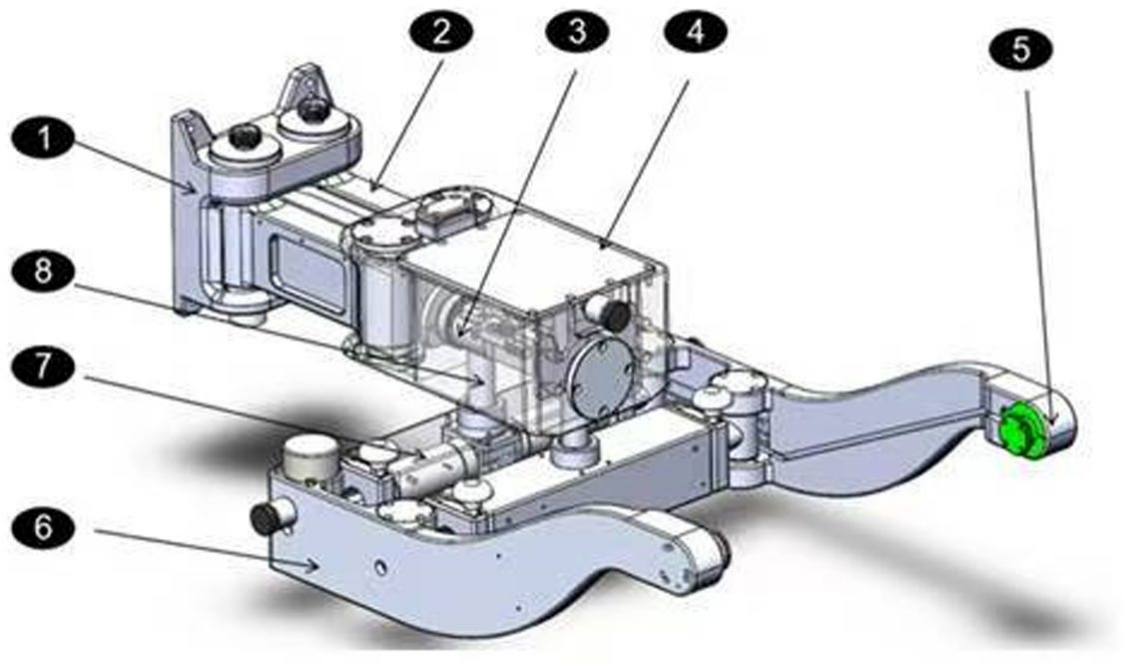
The NI controller.

The NI controller (shown in Figure 3) was used to get all the data values by DI, DO and serial modules. The data acquisition system (DAQ) is used to collect the pelvis system’s motion value, like rotation angle or lateral movement displacement. The NI controller is connected to the DAQ unit for data collection and interaction. The servo motor module communicates with the NI controller to realize the motor monitor and control. The user interface runs on a Windows machine and connected with the real time NI controller, so the VR system is. This distributed control structure reduces the length of the analog cables between the sensors and the analog to digital converters, and improves the signal to noise ratio. Furthermore, it allows to run a lower level control loop at a suitable update rates(10 millisecond).

Hip unloading perturbation training offers unique flexibility and adaptability compared to whole-body support systems or robot-assisted training. Not only does it improve training flexibility and adaptability, but by strengthening core muscle group training, it effectively enhances the patient’s balance ability and gait quality. This makes the hip unloading device particularly suitable for highly simulated natural gait and coping with sudden disturbances in rehabilitation training. Therefore, the device not only provides a training environment that conforms to physiological gait but also comprehensively enhances rehabilitation outcomes by strengthening key muscle group activities, expected to show greater therapeutic potential in stroke rehabilitation (13).

Additionally, perturbation training can increase the speed of balance recovery by strengthening muscle strength and reflex responses. This method continuously challenges the coordination and strength of muscle groups, enhancing their ability to contract reflexively, which is crucial for quickly generating the power needed to respond to balance disturbances (14).

Integrating perturbation training with traditional rehabilitation methods offers a comprehensive treatment approach, improving patients’ ability to manage daily activities and enhancing their quality of life (15). Therefore, it is recommended to incorporate perturbation training early in the rehabilitation process to leverage the period of high neural plasticity for optimal recovery.

Previous studies on hip unloading walking training are limited, with a few finding that such training effectively improves walking function in post-stroke patients, but more evidence is needed to verify its therapeutic effects. Perturbation training is an important means to improve balance function, commonly used to improve the balance function of elderly or Parkinson’s patients (16). Whether combining hip unloading with perturbation training can further enhance walking and balance functions in stroke patients is worth further study.

This study proposes a new strategy for the rehabilitation of walking function in post-stroke patients using hip unloading walking training combined with perturbation training, analyzing the improvement in walking and balance functions after training, aiming to explore new strategies for the rehabilitation of walking function in stroke patients.

## 2 Methods

### 2.1 Sample size calculation

Based on the results of a pilot study: the control group (*n* = 18) had a pre-treatment Berg score of 21.33 ± 11.36 and a post-treatment score of 28.50 ± 9.89; the experimental group (*n* = 15) had a pre-treatment Berg score of 23.89 ± 8.51 and a post-treatment score of 28.50 ± 4.67. Using an independent sample t-test to estimate sample size, assuming a power of 0.8, *α* = 0.017, μ1 = 5, μ2 = 3, calculations via PASS14.0.1 determined that 23 participants per group were needed. The participants were randomly assigned to three groups (hip unloading gait group, hip unloading with perturbation gait group, and control group) in a 1:1:1 ratio. Considering a dropout rate of approximately 30% and recruitment difficulties, a total of 30 participants were planned to be enrolled in each group to ensure an adequate sample size for data analysis.

### 2.2 Demographic information

This study was approved by the Ethics Committee of Beijing Tsinghua Changgung Hospital and involved 30 stroke patients aged 18–70 years treated at the hospital since 2021. Patients were matched by age and gender before being randomly assigned. Inclusion criteria included patients diagnosed according to the 1995 revision of the Diagnostic Points for Various Cerebrovascular Diseases by the Chinese Medical Association, confirmed by CT or MR imaging as having either a cerebral infarction or hemorrhage, with: (1) stable vital signs; (2) first-time occurrence; (3) within 6 months of onset; (4) Brunnstrom Stage III to IV; (5) Holden’s Walking Grade 3; (6) lower limb muscle tone of ≤2; (7) informed consent from a guardian. Exclusion criteria were: (1) unstable condition; (2) severe speech and cognitive impairments affecting training; (3) accompanying severe cardiopulmonary, liver, or kidney dysfunction precluding trial participation; (4) osteoarticular diseases; (5) visual or hearing impairments and motion sickness; (6) other neurological diseases such as Parkinson’s or epilepsy.

### 2.3 Study design

**Figure 4:**
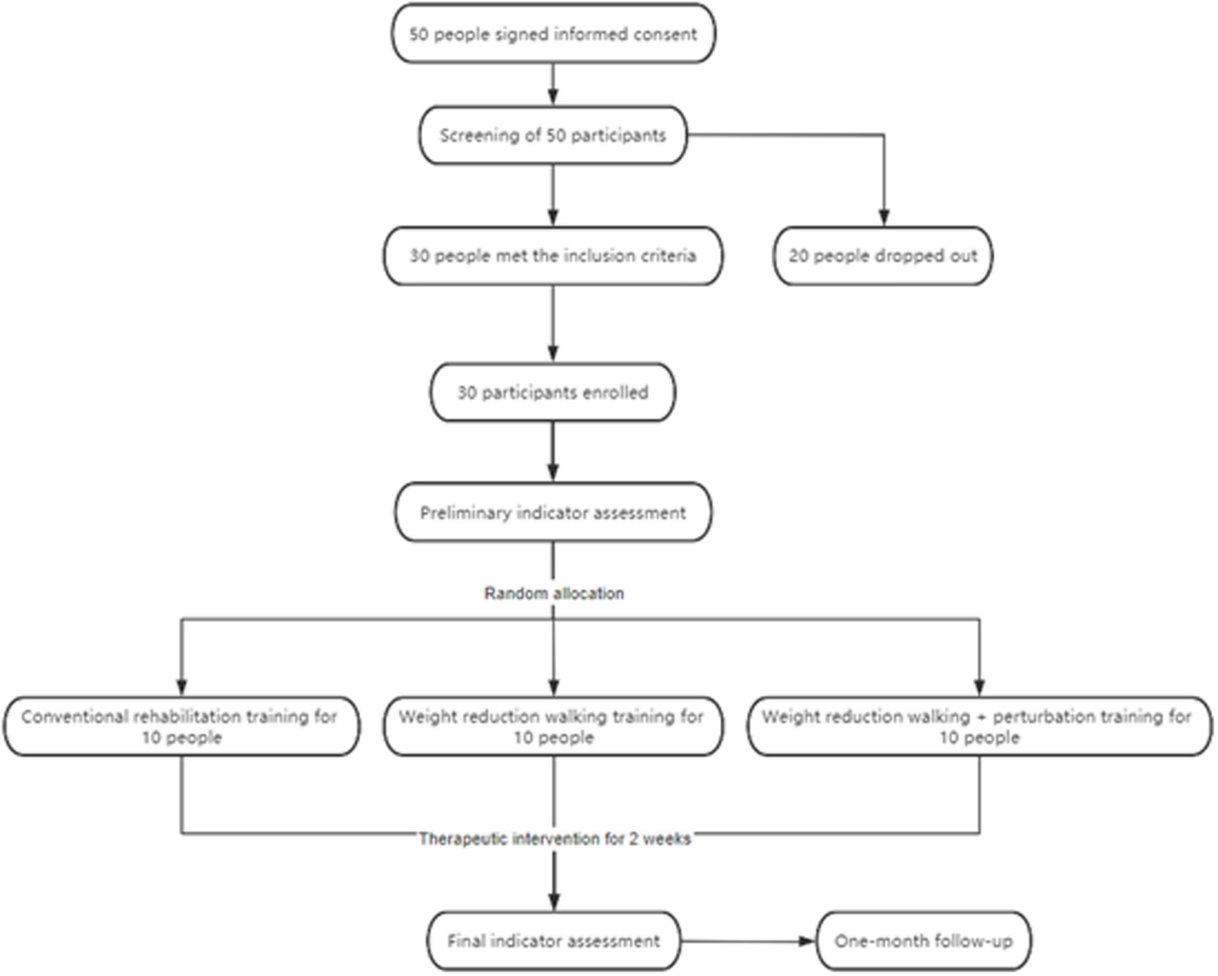
Flowchart of the trial.

**Figure 5:**
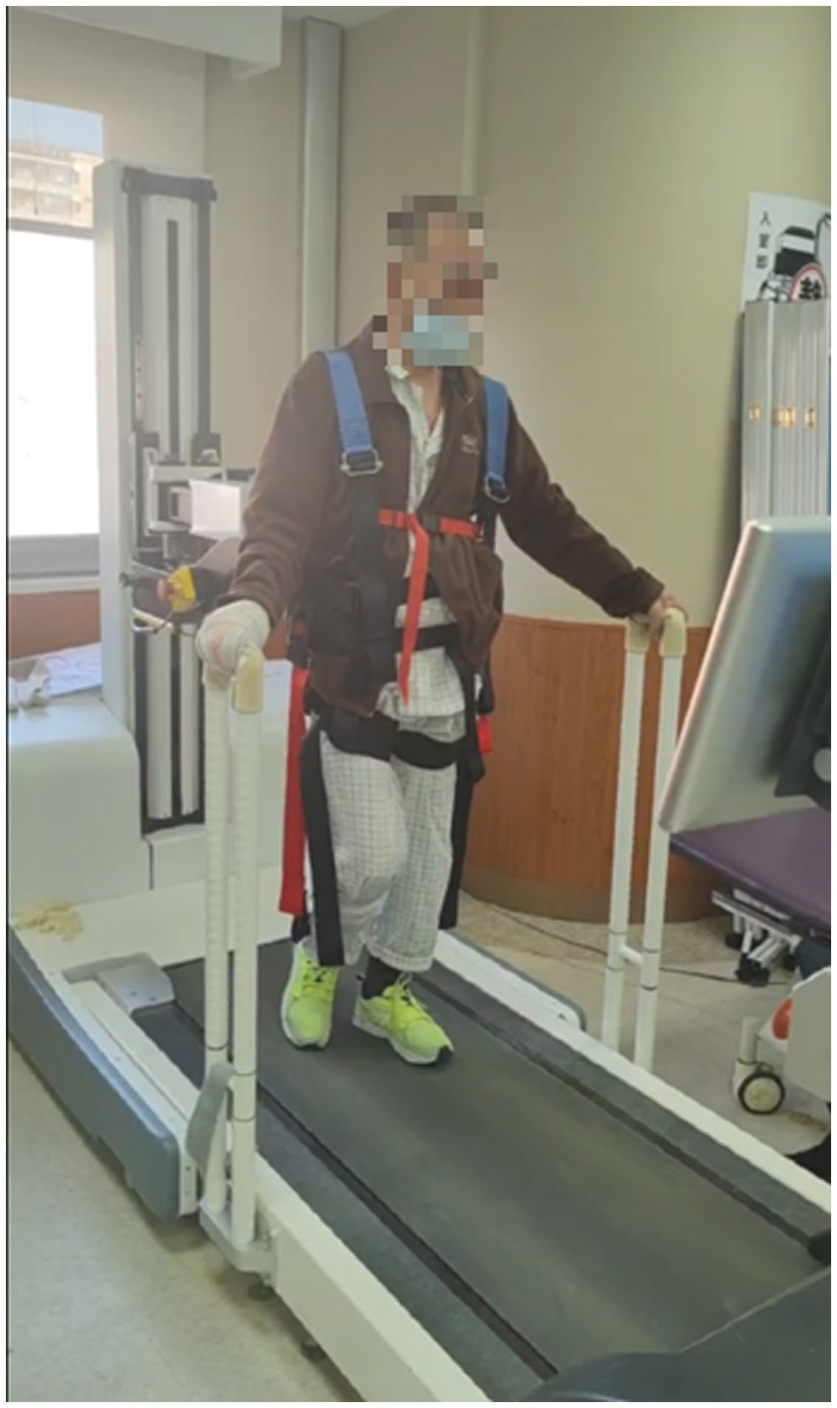
Participants in the Unloading Perturbation Group practicing standing and walking on electromechanical equipment.

Sociodemographic characteristics included age and gender. The overall screening and enrollment of the patients and the study plan are illustrated in Figure 4. The stroke patients included in this study were all ischemic stroke patients, and the laterality of the stroke affected the contralateral limb. This means that the limb on the opposite side of the affected cerebral hemisphere exhibited dysfunction. Specific demographic information (such as age, gender, etc.) has been compiled into a table, but will not be detailed here; the table is presented as Table 1. The 30 stroke patients were randomly divided into three groups: the hip unloading walking group (unloading group, *n* = 10), the hip unloading plus perturbation walking group (unloading perturbation group, *n* = 10), and the control group (*n* = 10). Treatment modalities provided were as follows:

1. *Control group*: Conventional rehabilitation training twice daily, including muscle strength training, core training, stepping exercises, parallel bar walking, and walking with assistance, each session lasting 30 min for 10 days. This was supplemented with conventional walking training for 20 min per session, once a day, 5 days a week for 2 weeks.
2. *Hip unloading walking group*: Conventional rehabilitation training supplemented with hip unloading walking training. Unloading sessions lasted 20 min each, once daily, 5 days a week, over a two-week course. Initial unloading was set at 30% of hip load, which was gradually reduced as patients adapted (17).
3. *Hip unloading perturbation group*: Conventional rehabilitation training plus hip unloading and perturbation training. Training sessions lasted 20 min each, once daily, 5 days a week, totaling 2 weeks.
4. *Perturbation training*: Patients underwent 20 forward perturbations, 20 backward perturbations, and 40 bidirectional perturbations (forward and backward) while standing on a treadmill. Forward perturbations were conducted at 0.15–0.2 m/s, and backward at 0.20–0.25 m/s. The setup for the unloading perturbation training is shown in Figure 5.

**Table 1:** Statistical table of stroke patient information

		Gender	Age	The type of stroke	The laterality of stroke
Control (*n* = 10)	1	Male	63	Ischemic	The Right cerebral hemisphere
	2	Female	58	Ischemic	The left cerebral hemisphere
	3	Female	56	Ischemic	The Right cerebral hemisphere
	4	Male	76	Ischemic	The left cerebral hemisphere
	5	Male	59	Ischemic	The Right cerebral hemisphere
	6	Male	67	Ischemic	The left cerebral hemisphere
	7	Female	59	Ischemic	The left cerebral hemisphere
	8	Male	59	Ischemic	The Right cerebral hemisphere
	9	Male	47	Ischemic	The left cerebral hemisphere
	10	Female	68	Ischemic	The left cerebral hemisphere
Unloading (*n* = 10)	11	Male	68	Ischemic	The left cerebral hemisphere
	12	Male	71	Ischemic	The Right cerebral hemisphere
	13	Male	57	Ischemic	The left cerebral hemisphere
	14	Male	55	Ischemic	The left cerebral hemisphere
	15	Female	68	Ischemic	The Right cerebral hemisphere
	16	Female	78	Ischemic	The left cerebral hemisphere
	17	Male	67	Ischemic	The Right cerebral hemisphere
	18	Male	57	Ischemic	The left cerebral hemisphere
	19	Male	75	Ischemic	The Right cerebral hemisphere
	20	Male	66	Ischemic	The left cerebral hemisphere
Perturbation (*n* = 10)	21	Female	78	Ischemic	The Right cerebral hemisphere
	22	Male	43	Ischemic	The left cerebral hemisphere
	23	Male	48	Ischemic	The left cerebral hemisphere
	24	Male	55	Ischemic	The left cerebral hemisphere
	25	Male	75	Ischemic	The Right cerebral hemisphere
	26	Male	53	Ischemic	The left cerebral hemisphere
	27	Female	72	Ischemic	The Right cerebral hemisphere
	28	Male	33	Ischemic	The left cerebral hemisphere
	29	Male	49	Ischemic	The Right cerebral hemisphere
	30	Male	63	Ischemic	The left cerebral hemisphere

Assessments using various scales were conducted before and after the two-week treatment period. After the completion of scale assessments, patients were followed up.

Follow-up plan: Pre-rehabilitation, 10 days post-treatment, and 1-month follow-up.

### 2.4 Assessment plan

Primary outcome measures for rehabilitation assessment:

1. *Berg balance scale (BBS)*: Used to evaluate an individual’s balance ability.
2. *10-meter walk test (10MWT, m/s)*: Measures walking speed over a short distance.

Secondary outcome measures:

1. *Simplified Fugl-Meyer assessment for lower extremity (FMA)*: Specifically assesses motor function and flexibility in hemiplegic limbs.
2. *Functional ambulation category (FAC)*: Evaluates mobility and flexibility in hemiplegic patients.
3. *Three-dimensional gait analysis*: Collects gait parameters, comfortable walking speed, and the percentage of support phase on the affected side.
4. *Timed up and go test (TUGT)*: Assesses the time it takes for a patient to stand up, walk, and sit down again, reflecting walking safety and speed.
5. *Brunnstrom stage*.
6. *Lower limb muscle tone rating*: Evaluates the tension in lower limb muscles.

These assessments have demonstrated strong interpretability and test–retest reliability in evaluating balance during the rehabilitation walking process for stroke patients.

Demographic Information and Pre- and Post-Training Score Changes as shown in Table 2. After controlling for the effects of gender and age across groups, there were no significant differences in Holden walking, Brunnstrom stages, FMA, and lower limb muscle tone prior to training.

**Table 2:** Demographic information and pre- and post-training score changes.

				Before				After			
		Gender	Age	Brunnstrom Stages	Holden Walking Grade	Lower Limb Muscle Tone Rating	FMA	Brunnstrom Stages	Holden Walking Grade	Lower Limb uscle Tone Rating	FMA
Control(*n* = 10)	1	Male	63	4	2	1+	18	4	2	1	21
	2	Female	58	5	4	1	30	5	4	0	31
	3	Female	56	4	1	2	24	5	3	0	32
	4	Male	76	5	2	1	28	5	3	0	33
	5	Male	59	4	2	1	28	5	3	0	33
	6	Male	67	4	2	1	26	5	2	1	27
	7	Female	59	3	1	2	24	4	1	1	27
	8	Male	59	6	3	0	30	6	4	0	32
	9	Male	47	4	3	0	33	5	4	0	34
	10	Female	68	4	3	1	29	4	3	0	29
Unloading(*n* = 10)	11	Male	68	3	0	1+	16	4	2	1	20
	12	Male	71	4	3	1	22	4	3	1	23
	13	Male	57	3	2	1	21	3	3	1	23
	14	Male	55	5	2	1+	26	5	2	0	30
	15	Female	68	5	3	1+	21	5	4	0	29
	16	Female	78	3	4	1+	25	4	4	0	26
	17	Male	67	4	3	0	21	4	3	0	25
	18	Male	57	4	2	1+	23	5	2	1	27
	19	Male	75	3	2	1+	21	4	3	1	22
	20	Male	66	4	3	1+	23	5	4	0	24
Perturbation(*n* = 10)	21	Female	78	4	3	1+	19	4	4	1	29
	22	Male	43	4	4	2	23	4	3	0	26
	23	Male	48	6	5	0	34	6	5	0	34
	24	Male	55	4	3	1+	22	4	4	0	25
	25	Male	75	4	2	2	25	4	3	0	27
	26	Male	53	3	2	1+	15	4	3	1	27
	27	Female	72	3	2	0	20	3	2	0	23
	28	Male	33	6	5	0	30	6	5	0	33
	29	Male	49	4	3	2	31	5	3	1	33
	30	Male	63	5	2	0	28	5	3	0	32

### 2.5 Statistical analysis

Data for this study were processed using IBM SPSS Statistics 21.0. Quantitative data were tested for normality. Normally distributed data were presented as mean ± standard deviation and compared between groups using Analysis of Variance (ANOVA). For data not normally distributed, the Mann–Whitney U test was employed, and Fisher’s exact test was used when any expected cell counts were less than five. Qualitative data were presented as proportions and compared using the Chi-square test. Multiple comparisons were adjusted using the Bonferroni correction as appropriate. Paired *t*-tests were used for within-group comparisons, and one-way ANOVA was employed for comparisons of metric data between the three groups. A *p*-value of <0.05 was considered statistically significant.

## 3 Results

### 3.1 Primary outcomes

In our comparative study, we analyzed the changes in balance ability and 10-Meter Walk Test (10MWT) scores among three patient groups following different interventions.

#### 3.1.1 Berg balance scale

Prior to treatment, the initial assessments for the three groups were as follows:

Control Group, Unloading Group, and Perturbation Group showed changes in average scores from pre to post-training as follows: The Control Group increased from 41.3 ± 8.30 to 43.9 ± 9.04, the Unloading Group from 30.2 ± 9.33 to 32.3 ± 10.55, and the Perturbation Group significantly from 35.7 ± 14.54 to 41.6 ± 11.68. In balance assessments, all groups demonstrated some improvement in balance capabilities, but the improvement in the Perturbation Group (5.9 ± 2.85) was significantly higher than in the Control Group (2.6 ± 0.74) and the Unloading Group (2.1 ± 1.22) (Table 3).

**Table 3:** Pre- and post-training comparison on the berg balance scale for control, unloading, and perturbation groups.

Group	Pre-training	Post-training	Difference
Control (*n* = 10)	41.30 ± 8.30	43.90 ± 9.04	2.60 ± 0.74
Unloading (*n* = 10)	30.20 ± 9.33	32.30 ± 10.55	2.10 ± 1.22
Perturbation (*n* = 10)	35.70 ± 14.54	41.60 ± 11.68	5.90 ± 2.85

In this study, the Berg Balance Scale was employed to comprehensively analyze the effects of three different interventions on patient groups by comparing before and after scores. The results of the difference analysis are presented in Table 4, which evaluates their impact on balance abilities.

**Table 4:** Comparison and statistical significance of differences in berg balance scale scores.

Comparison	Mean difference	Standard error	Significance (P)	95% Confidence interval	
				Lower limit	Upper limit
Control vs. Unloading	0.50	1.55	0.749	−2.68	3.68
Perturbation vs. Control	3.30	1.55	0.042^*^	0.12	6.48
Perturbation vs. Control	3.80	1.55	0.021^*^	0.32	6.98

The results demonstrated significant enhancements in balance abilities among participants who underwent specific perturbation training. Statistically, the perturbation group exhibited notable improvements compared to the control group, with a difference of 3.3 and a 95% confidence interval ranging from 0.1203 to 6.4797 (*p* = 0.042). Similarly, the perturbation group showed significant gains over the weight reduction group, with a difference of 3.8 and a 95% confidence interval from 0.6203 to 6.9797 (*p* = 0.021).

The post-training difference analysis of balance scale scores across the three groups, detailed in Table 4, shows that the perturbation group’s balance abilities significantly surpassed those of both the control group (*p* = 0.042 < 0.05) and the weight reduction group (*p* = 0.021 < 0.05). This indicates that perturbation training may be more effective for improving patient balance abilities.

**Figure 6:**
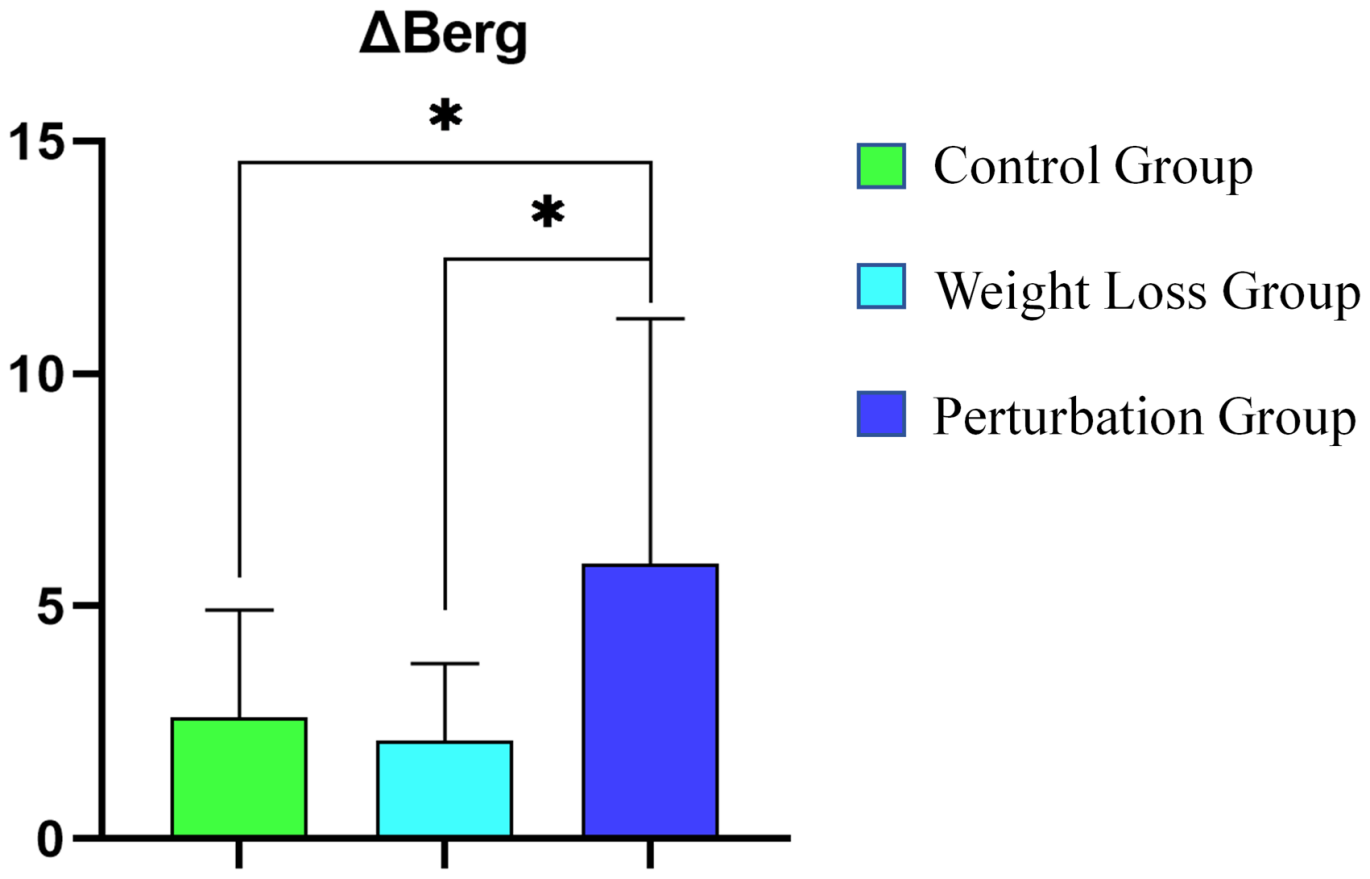
Statistical Comparison of ΔBerg Among Three Groups (*denote statistical significance).

Figure 6 shows a statistical comparison of the changes in Berg Balance Scale (ΔBerg) among three groups: the control group, the weight loss group, and the perturbation group. The results demonstrate that the ΔBerg values for the perturbation group are significantly higher than those for both the control and weight loss groups (*p* < 0.05). Specifically, the ΔBerg value for the control group is approximately 5, the weight loss group has a slightly lower ΔBerg value, and the perturbation group has a ΔBerg value of 14.9. This indicates that the improvement in Berg Balance Scale scores is markedly greater in the perturbation group compared to the other two groups.

#### 3.1.2 10-meter walk test

In terms of 10MWT scores, all groups showed improvement post-training as seen in Table 5. The average scores for the Control Group decreased from 29.36 ± 17.06 to 27.66 ± 16.00, the Unloading Group from 27.73 ± 9.08 to 26.42 ± 9.02, and the Perturbation Group significantly from 34.26 ± 16.08 to 27.15 ± 12.82. Although all groups improved, the Perturbation Group’s improvement (−7.11 ± 3.26) far exceeded that of the Control Group (−1.7 ± 1.06) and the Unloading Group (−1.31 ± 0.06), further confirming the potential advantages of perturbation training in enhancing walking efficiency and speed.

**Table 5:** Pre- and post-training 10MWT scores comparison.

Group	Pre-training	Post-training	Difference
Control (*n* = 10)	29.36 ± 17.06	27.66 ± 16.00	−1.70 ± 1.06
Unloading (*n* = 10)	27.73 ± 9.08	26.42 ± 9.02	−1.31 ± 0.06
Perturbation (*n* = 10)	34.26 ± 16.08	27.15 ± 12.82	−7.11 ± 3.26

Following multiple comparisons using the Least Significant Difference (LSD) method, the analysis results are presented in Table 6.

**Table 6:** LSD multiple comparison analysis results for 10MWT.

Comparison	Mean difference	Standard error	Significance (P)	95% Confidence interval	
				Lower limit	Upper limit
Control vs. Unloading	−0.40	2.41	0.87	−5.35	4.56
Perturbation vs. Control	−5.41	2.41	0.03^*^	−10.36	−0.46
Perturbation vs. Control	−5.80	2.41	0.02^*^	−10.76	−0.85

The findings reveal that the perturbation group exhibited notable enhancements in the 10-meter walk test at the post-test compared to both the control and weight reduction groups (difference from control group = −5.411, *p* = 0.033; difference from weight reduction group = −5.806, *p* = 0.023). This significant improvement underscores the critical role of perturbation interventions in boosting walking efficiency. The comparison between the control and weight reduction groups showed no statistical significance (difference = 0.395, *p* = 0.871).

The significant results for the perturbation group, with 95% confidence intervals not including zero (ranging from 0.4595 to 10.3625 against the control group, and from 0.8545 to 10.7575 against the weight reduction group), validate the statistical relevance and underline the clinical effects. LSD.

**Figure 7:**
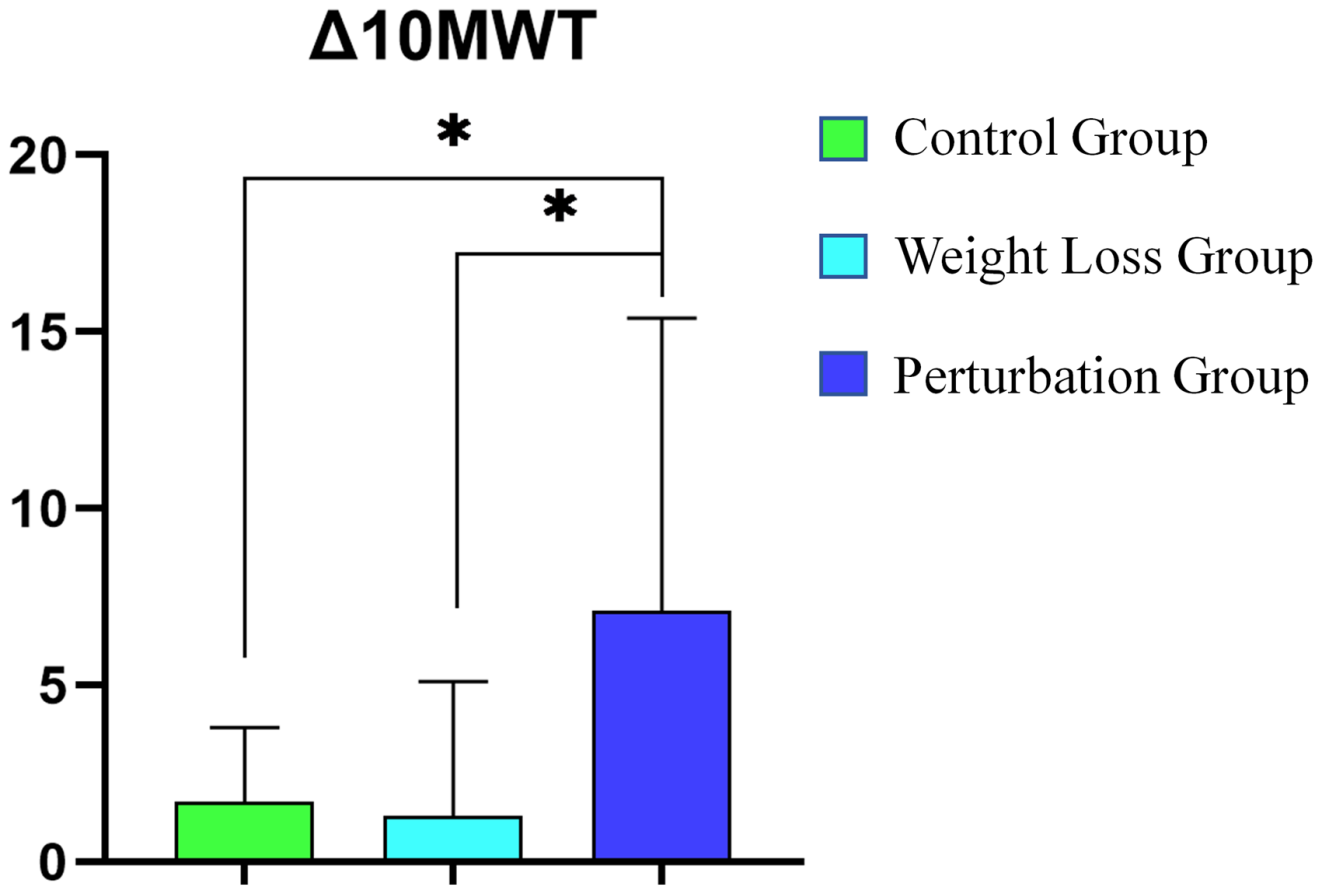
Statistical Comparison of Δ10MWT Among Three Groups (*denote statistical significance).

Figure 7 presents a statistical comparison of the changes in the 10-Meter Walk Test (Δ10MWT) among three groups: the control group, the weight loss group, and the perturbation group. The horizontal axis represents these groups, while the vertical axis displays the Δ10MWT values. The results indicate that the Δ10MWT changes for the control and weight loss groups are minimal, with values of approximately 2 and 1, respectively. In contrast, the perturbation group shows a significant increase in Δ10MWT, with a value nearing 15.

### 3.2 Secondary outcomes

In this study, we quantitatively evaluated the effects of interventions across three groups using a comprehensive set of assessment tools. These included Brunnstrom staging, the Holden Walk Test, lower limb muscle tone, the Fugl-Meyer Assessment (FMA) for lower limb function, the Functional Ambulation Category (FAC), and the Timed Up and Go Test (TUGT).

For the analysis of multiple comparisons, the Least Significant Difference (LSD) method was utilized to assess the scores from the Timed Up and Go Test (TUGT). As indicated in Table 7, significant differences in average TUGT scores were observed between the perturbation group and both the control group and the weight reduction group.

**Table 7:** Results of the LSD multiple comparison analysis for TUGT scores.

Comparison	Mean difference	Standard error	Significance (P)	95% Confidence interval	
				Lower limit	Upper limit
Control vs. Unloading	−5.54	4.12	0.191	−14.02	2.94
Perturbation vs. Control	−5.58	4.01	0.176	−13.83	2.67
Perturbation vs. Control	−11.12*	4.12	0.01*	−19.60	−2.64

The analysis between the perturbation group and the control group revealed an average difference of-5.581 with a standard error of 4.01464; however, this difference was not statistically significant (*p* = 0.176) with a 95% confidence interval from-13.8332 to 2.6712. This indicates that despite a mean decrease of 5.581 points in the perturbation group’s scores, the result was not statistically meaningful due to the large standard error and wide confidence interval. In comparison, the control group versus the weight reduction group demonstrated an average difference of-5.54 with a standard error of 4.12465, which also lacked statistical significance (*p* = 0.191) with a confidence interval spanning from-14.0183 to 2.9383, indicating no meaningful differences in TUGT scores between these groups.

Conversely, a notable average difference of-11.12100 (standard error 4.12465, *p* = 0.012), with a 95% confidence interval that excludes zero (ranging from-19.5993 to-2.6427), was observed between the perturbation and weight reduction groups. This substantial decrease in TUGT scores for the perturbation group underlines its potential efficacy in improving lower limb functionality and walking efficiency.

Similarly, the changes in the Fugl-Meyer Assessment (FMA) scores were evaluated using the Least Significant Difference (LSD) analysis, as shown in Table 7. The comparison between the control and weight reduction groups showed a minimal average difference of-0.1 with a standard error of 1.301, which was statistically insignificant (*p* = 0.939) with a 95% confidence interval ranging from-2.7694 to 2.5694. In the comparison between the perturbation and control groups, an average difference of 1.3 with a standard error of 1.301 was observed. This difference, with a *p*-value of 0.327 and a confidence interval extending from −1.3694 to 3.9694, also failed to achieve statistical significance. Lastly, the comparison of the perturbation group to the weight reduction group yielded an average difference of 1.2, standard error 1.301, p-value of 0.365, and a confidence interval from −1.4694 to 3.8694. No significant differences were noted between these groups either (Table 8).

**Table 8:** Results of the LSD multiple comparison analysis for FMA score differences.

Comparison	Mean difference	Standard error	Significance (P)	95% Confidence Interval	
				Lower limit	Upper limit
Control vs. Unloading	−0.10	1.30	0.94	−2.77	2.57
Perturbation vs. Control	1.30	1.30	0.33	−1.37	3.97
Perturbation vs. Control	1.20	1.30	0.36	−1.47	3.87

## 4 Discussion

### 4.1 Primary and secondary outcomes

In this study, we evaluated the impact of different rehabilitation interventions on balance and walking efficiency among three patient groups using the Berg Balance Scale and the 10-Meter Walk Test (10MWT), two widely recognized measurement tools (18). Post-intervention results showed enhancements in both balance and walking abilities across all groups, with the perturbation group demonstrating significant improvements in Berg Scale scores. These gains were consistent with the observed enhancements in walking efficiency during the 10MWT, highlighting the effectiveness of perturbation training in simulating real-life balance challenges and enhancing functional mobility. These findings align with prior research and support the hypothesis that perturbation training is beneficial for improving gait and balance by providing specific stimuli that enhance neural plasticity and motor skill learning (19).

Table 4 findings suggest that high-intensity, task-specific perturbation training is more likely to foster neural plasticity and enhance motor learning. Although no statistically significant balance improvements were observed in the weight reduction group compared to the control group (difference of-0.5, *p* = 0.749), this does not negate the potential benefits of weight reduction training under certain conditions.

Figure 1 statistical analysis confirms the significance of these differences, as denoted by the asterisks in the figure. Overall, the data suggest that the perturbation group achieved the most substantial enhancement in balance ability.

Table 6 multiple Comparison Analysis Results for 10MWT the non-significant enhancements observed in the control and weight reduction groups also hold some significance, suggesting that even in the absence of specific interventions, the inherent recovery processes in patients might contribute to functional improvements.

Figure 2 statistical analysis reveals a significant difference between the perturbation group and both the control and weight loss groups (*p* < 0.05), indicating that the perturbation group achieved a substantial improvement in the 10-Meter Walk Test. These findings underscore the effectiveness of perturbation training in enhancing walking ability.

Table 7 Results of the LSD Multiple Comparison Analysis for TUGT Scores the statistical analysis demonstrates significant distinctions in functional outcomes after treatment, particularly in FMA lower limb scores and TUGT performance, suggesting that the perturbation group’s intervention was uniquely effective compared to the control and weight reduction groups (*p* < 0.05).

Patients in the weight reduction group demonstrated improvements on certain scales, though these enhancements did not achieve the significant levels observed in the perturbation weight reduction group across all tests. Although the control group’s data did not show statistically significant changes, signs of improvement were evident in some scale tests. These observations suggest that standard interventions may still offer potential benefits to specific patients, though their effects are generally more modest compared to targeted intervention strategies.

Consolidating these evaluation results, while some statistical outcomes for the scales were not significant, all intervention groups experienced enhancements on these secondary indicators, with the perturbation group showing the most substantial improvements in the majority of tests. This pattern aligns with our primary findings, further endorsing the pivotal role of perturbation training in rehabilitation programs. Although improvements in the control and weight reduction groups were statistically inconsequential, the emerging positive trends should not be overlooked. These trends may indicate that even non-specific interventions can positively influence the natural recovery processes in patients.

The findings underscore that an integrated approach to interventions, particularly those incorporating perturbation training, may surpass traditional methods in improving lower limb functionality and overall motor coordination.

The modest improvements noted in the control group could reflect aspects of natural recovery or an adaptive response to the experimental protocol. Even these limited enhancements serve as important benchmarks for evaluating other therapeutic strategies.

The relatively smaller gains observed in the Berg Scale and 10MWT scores for the control and weight reduction groups may indicate inadequacies in the personalization of current rehabilitation programs. This underscores the need for future rehabilitation strategies to be more patient-centric, tailoring interventions to meet individual needs (20). The absence of statistically significant improvements in the weight reduction group does not negate its effectiveness in clinical settings but may point to potential benefits that have not been fully recognized across various clinical environments or patient subtypes (21).

Additionally, the study incorporated other essential metrics like the Fugl-Meyer Assessment (FMA) and the Functional Ambulation Category (FAC), covering motor function and balance, movement speed, as well as muscle function and tone. These tools provide a broader perspective on lower limb motor functions and walking capabilities (22–24).

In assessments such as the Timed Up and Go Test (TUGT), while improvements were noted in the control and weight reduction groups, the perturbation group showed the most substantial decrease in scores, achieving statistical significance compared to the weight reduction group. This reinforces the efficacy of perturbation training in enhancing motor functions, especially in terms of improving walking ability and reducing fall risk.

The improvements in secondary outcomes for the perturbation group align with the primary scale results, supporting the comprehensive role of perturbation training in enhancing overall patient motor functions (25).

This research underscores the significant benefits of perturbation training in boosting balance abilities and recommends integrating it into rehabilitation programs, particularly for patients with severe balance challenges. Clinically, perturbation training should be a component of routine treatment plans, especially targeting those with significant balance impairments (15). The findings also advocate for treatment plans that are tailor/ed to the specific needs of patients and clearly define their clinical application to maximize the effectiveness of interventions.

### 4.2 Mechanisms and applications of perturbation training

Perturbation training employs unstable environmental stimuli that compel patients to make rapid balance adjustments, effectively activating the central nervous system’s response mechanisms (15). This strategy primarily enhances reactive balance control, which is the ability of the central nervous system to mobilize various body parts swiftly in response to sudden balance disturbances (26). Specifically, the frequent disruptions and subsequent recoveries in balance can promote neural plasticity, aiding in the repair of damaged neural pathways or the creation of new connections, thus improving patients’ motor coordination and balance.

Studies have shown that perturbation training is particularly effective for stroke patients with severe balance disorders and should be considered a vital component of rehabilitation programs (5). To maximize outcomes, clinicians should tailor the training’s frequency, intensity, and duration based on personalized assessments to ensure it is conducted in the safest possible conditions.

In conclusion, perturbation training demonstrates significant benefits in rehabilitation medicine due to its unique physiological mechanisms, especially in enhancing the balance and walking functions of stroke patients. Future research should expand its application to different patient groups to more fully evaluate its clinical utility (27).

This study’s limitations include a small sample size and specific design constraints, which may affect the generalizability of the findings. Future research should increase the sample size and use more rigorous methodologies to validate these results and explore the rehabilitative effects of other interventions (28). Consideration of factors such as the frequency, duration, and specific patient responses to interventions will be crucial in future studies (29).

Overall, this research emphasizes the crucial role of perturbation training in rehabilitation, especially for improving balance and walking functions. By advancing research and refining practices, rehabilitation services can be enhanced, improving patients’ quality of life and functional independence. It is advisable to fully integrate perturbation training into future rehabilitation efforts and to further investigate the long-term effects of weight reduction training, employing a variety of assessment tools to comprehensively evaluate patients’ rehabilitation progress, thereby providing higher quality and more effective rehabilitation services.

## Data Availability

The raw data supporting the conclusions of this article will be made available by the authors, without undue reservation.
